# Revisiting two-stage models of chemically-induced cirrhosis-associated hepatocarcinogenesis in rodents: methodological and histopathological approaches

**DOI:** 10.1007/s10735-026-10899-9

**Published:** 2026-07-14

**Authors:** Antonio Rodrigues Bueno da Fonseca, Paulo Franco Cordeiro de Magalhães Junior, Luis Fernando Barbisan

**Affiliations:** 1 Department of Cellular and Molecular Biology, Institute of Biosciences of Botucatu, São Paulo State University (UNESP), Botucatu, SP Brazil; 2 Higher School of Health Sciences, Federal University of Amazonas (UFAM), Manaus, AM Brazil

**Keywords:** Chemically-induced liver fibrosis/cirrhosis, Hepatocarcinogenesis, Two stage rodent models, Histopathology

## Abstract

**Abstract:**

Repeated exposure to hepatotoxins such as carbon tetrachloride (CCl_4_), thioacetamide (TAA), or diethylnitrosamine (DEN) has been frequently used to induce fibrosis/cirrhosis in rodents. In this narrative review, we compared two-stage medium-term protocols using different CCl_4_ or TAA regimens in DEN-initiated rats and mice. In addition, we revisited our previous two-stage DEN/CCl_4_ or DEN/TAA rat models to compare fibrosis grade, the mean number, and the percentage of liver area occupied by hepatocellular lesions positive for placental glutathione S-transferase (GST-P). Thus, we evaluated two groups of male Wistar rats that received DEN (a single dose), as an initiator agent for liver carcinogenesis, followed by CCl_4_ or TAA regimens (multiple doses) until week 25. Both male Wistar groups presented lower body weight gain and higher liver weight, serum alanine aminotransferase (ALT) levels, hepatocyte proliferation, and a frank development of fibrosis/cirrhosis, GST-P-positive preneoplastic lesions, and liver tumors. However, DEN/TAA protocol in male Wistar rats was more effective as a fibrosis/cirrhosis-associated hepatocarcinogenesis model since TAA effectively accelerates liver disease progression and carcinogenesis. In addition, these chemical regimens have been prospectively studied as a suitable and effective protocol that resembles the corresponding human disease with various molecular and morphological outcomes. In conclusion, two-stage DEN/CCl_4_ or DEN/TAA rodent models are effective in mimicking the progression of fibrosis-associated hepatocarcinogenesis and are suitable for prophylactic or therapeutic investigations.

**Graphical abstract:**

**Figure:**
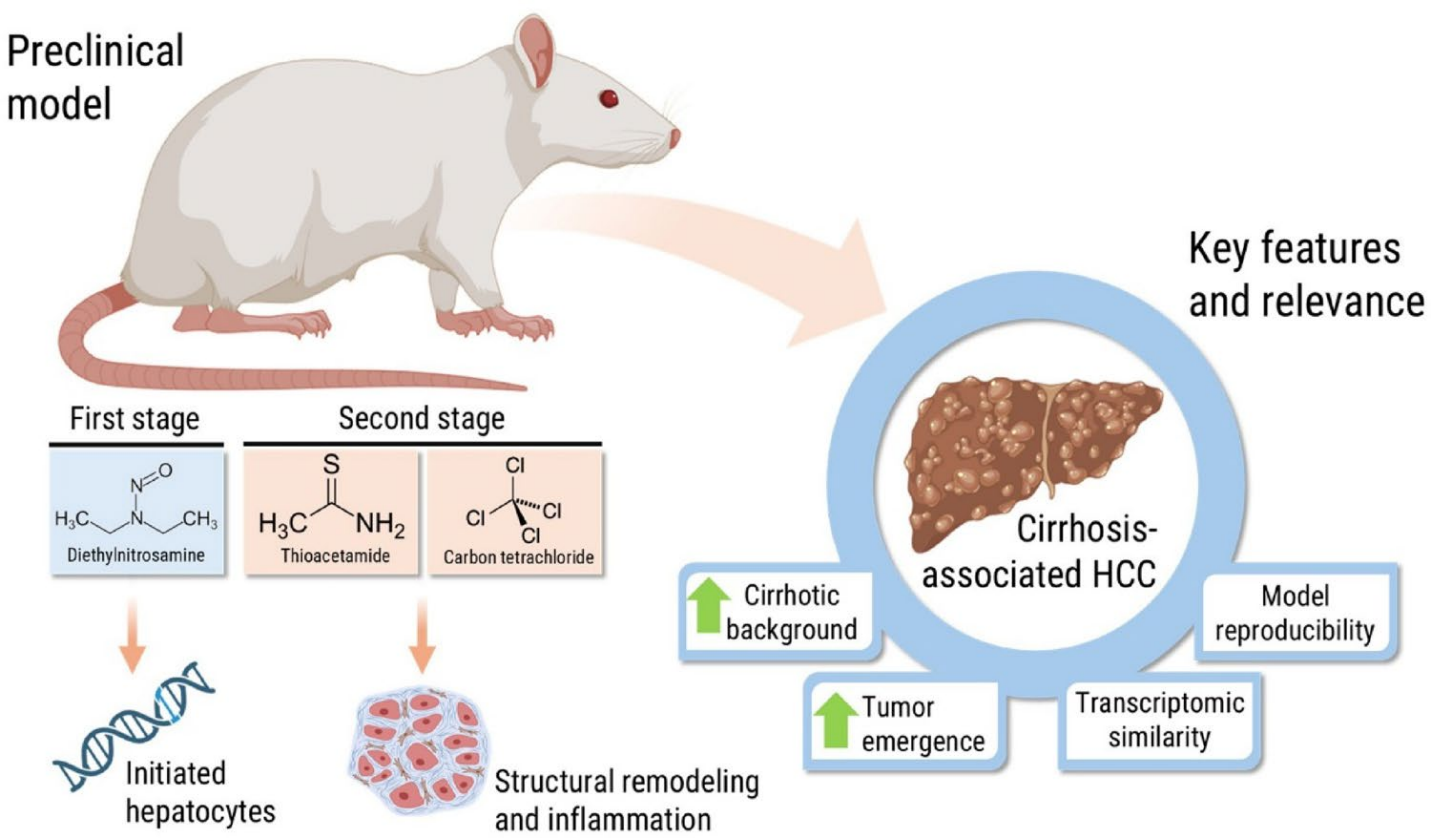

## Introduction

Recent worldwide-scope epidemiological studies regarding trends of chronic liver diseases highlight the major relevance of liver advanced fibrosis and its progressive and late-stage condition cirrhosis onset, estimated to affect 3.3 and 1.3%, respectively, with an alarming demographic focus on African (4.0 and 2.8%, respectively), European (3.4 and 1.0%, respectively), and North American (4.8 and 1.6%, respectively) continents (Huang et al. 2023; Zamani et al. 2025). Such findings also provide insights into a steady increase on liver fibrosis/cirrhosis prevalence over the past decade (0.8% before 2010 to 1.8% after 2016) (Huang et al. 2023). In this context, hepatocellular carcinomas (HCC) is one of the most aggressive liver diseases and the major causes of cancer-related mortality worldwide, showing high malignancy and poor prognosis. Liver cirrhosis is the major risk factor associated with 80–90% HCC cases and a major leading cause for liver transplantation (Ginès et al. 2021; Huang et al. 2023; Devarbhavi et al. 2023; Zamani et al. 2025). This main risk factor is a complex process characterized by progressive fibrogenesis and continuous hepatocyte necrosis/regeneration cycles, leading to the formation of several regenerative nodules, portal hypertension, and liver failure as an end-stage of this chronic liver disease (Rumgay et al. 2022; Huang et al. 2023). Multiple macro and micronodular regenerative lesions are observed during the progression of liver cirrhosis, mainly induced by a persistent insult such as chronic hepatitis B and C virus (HBV and HCV) infections, ethanol abuse, and metabolic dysfunction-associated steatotic liver disease (MASLD) establishment (Lee et al. 2015; Putignano and Gustot 2017; Ginès et al. 2021). The continuous damage of liver parenchyma activates major wound-healing pathways that remodel the spatial organization of hepatic parenchyma through the extracellular matrix (ECM) synthesis, enabling the emergence of preneoplastic foci and neoplastic lesions (Hammerich and Tacke 2023). Within this microenvironment, dysplastic nodules, hepatocellular adenomas (HCA), and HCC can emerge (Hammerich and Tacke 2023; Devarbhavi et al. 2023; Umetsu and Kakar 2023; Wen and Kakar 2024).

Several chemically induced rodent models for fibrosis/cirrhosis-associated hepatocarcinogenesis have been widely applied in translational research since they mimic some of the human disease features, including low (histological, clinical, and biochemical findings) and high (molecular) resolution outcomes (Furtado et al. 2014; Yanguas et al. 2016; Faccioli et al. 2022; Romualdo et al. 2017, 2021, 2023; Hu et al. 2024). In rodent models, chronic administration of hepatotoxic drugs such as diethylnitrosamine (DEN), carbon tetrachloride (CCl_4_), thioacetamide (TAA), isolated or in combination, has been commonly used to induce fibrosis/cirrhosis-associated hepatocarcinogenesis (Furtado et al. 2014; Yanguas et al. 2016; Romualdo et al. 2017, 2021, 2023; Hu et al. 2024). Thus, some two-stage rodent protocols have used the association between DEN (i.e., a genotoxic agent administered in single or multiple applications) and CCl_4_ or TAA regimens (i.e., inducers of liver injury by oxidative stress and administered in multiple applications) as effective approaches to mimic the progression of fibrosis/cirrhosis-associated hepatocarcinogenesis with a high incidence of liver tumors in medium-term time-points (Furtado et al. 2014; Romualdo et al. 2017; Dwivedi and Jena 2020).

In DEN-initiated mice and rats, CCl_4_ and TAA act as fibrosing and tumor promoting agents that induces a marked number of regenerative nodules separated by fibrous septa, and associated with activated hepatic stellate cells (HSCs), oxidative stress, and inflammation, as well as the development of foci of altered hepatocytes (FAH) and hyperplastic nodules—as preneoplastic lesions—and tumors such as HCA, HCC, cholangiomas and cholangiocarcinomas (Furtado et al. 2014; Romualdo et al. 2017; Dwivedi and Jena 2020; Memon et al. 2020; Uehara et al. 2021). In a mouse DEN-induced HCC model, several single-nucleotide variants were detected that might be associated with the activation of oncogenic hotspots, such as *Hras*, *Braf*, *Apc*, and *Tp53*, which were described as the main canonical drivers of HCC (Connor et al. 2018). In previous studies, the wide-spectrum assessment of CCl_4_ and TAA models showed the main role of branch-chain amino acids, inflammation-related, and ECM remodeling pathways at both RNA and protein levels, which are consistent with prominent tumor biology on liver fibrosis/cirrhosis onset in patients (Teck et al. 2004; Connor et al. 2018; Zhang et al. 2024). However, a comparative evaluation of these models regarding their similarity to the corresponding human late-stage disease is still elusive.

In the past years, the advent of new molecular biology approaches has introduced new layers of complexity to the understanding of the hepatocarcinogenesis process in different experimental models, making it urgent to clearly understand the high-resolution insights of HCC biology (Hasin et al. 2017; Petrenko et al. 2024). This paradigm shift underscores the importance of integrating these high-resolution molecular data when evaluating the translational relevance of experimental models (Hasin et al. 2017). Thus, in this review, we revisited and compared the main findings of two-stage protocols for induction of fibrosis/cirrhosis-associated hepatocarcinogenesis—especially DEN-initiated and CCl_4_/TAA-promoted bioassays—including data of our previous studies. Addressing this gap is essential for improving the translational relevance of these models and understanding how different mechanisms of chronic liver injury influence hepatocarcinogenesis. In addition, preclinical DEN plus CCl_4_/TAA models are suitable tools used to evaluate the effectiveness of preventive and therapeutic interventions.

## Rodent models of fibrosis-associated hepatocarcinogenesis using a single agent

DEN is a classical hepatocarcinogen that is mainly metabolized in the liver by cytochrome P450 isoenzyme 2E1 (CYP2E1), generating reactive oxygen species (ROS) and the nucleophilic ethyldiazonium ions, which induce DNA damage and genomic instability, with the appearance of initiated hepatocytes that present potential for clonal expansion into early carcinogenesis (Tolba et al. 2015; Gao et al. 2018; Memon et al. 2020). The administration of single or multiple DEN injections in rodents, at different doses and ages, has variable efficacy and efficiency in HCC development (Vesselinovitch et al. 1984; Tolba et al. 2015; Romualdo et al. 2021, 2023). Specially in mouse protocols, DEN administration can be performed in infant/neonate animals for a more effective initiation of hepatocarcinogenesis (Vesselinovitch et al. 1984, 1987; Tolba et al. 2015; Memon et al. 2020; Schulien and Hasselblatt 2021), since an elevated hepatocellular proliferation rate in developing liver facilitates the clonal expansion of DEN-initiated hepatocytes and, hence, tumor emergence. Thus, the main outcome of these infant/neonate mouse models is earlier latency for the development of preneoplastic and neoplastic lesions when compared to DEN initiation occurring in the liver of adult mice (Schulien and Hasselblatt 2021). In rodent protocols (mainly using males), most studies use multiple DEN administrations (moderate to high doses) for induction of cirrhosis and HCC; however, liver toxicity, weight loss, and animal mortality are common in these protocols (Tolba et al. 2015; Memon et al. 2020; Kurma et al. 2021).

CCl_4_ is a hepatotoxicant also metabolized by the CYP2E1 to the highly reactive oxygen trichloromethyl (*CCl_3_) and trichloromethyl peroxyl (*OOCCl_3_) radicals that induce oxidative stress and liver injury (Weber et al. 2003; Romualdo et al. 2018). Chronic CCl_4_ use (intragastrical or intraperitoneal administrations) causes hepatocyte death with compensatory cell regeneration, inflammation, and fibrosis/cirrhosis, associated with HSC activation and increased collagen synthesis (Weber et al. 2003; Yanguas et al. 2016; Romualdo et al. 2018). Although this classical hepatotoxin is rarely used in isolation as a model for HCC development, the induction of rodent hepatic tumors by CCl_4_ following various routes of exposure is dependent upon the dose (concentration and exposure duration), with high HCC burden only occurring at cytotoxic exposure levels in long-term assays (2 years) (Cohen et al. 2023).

TAA causes hepatotoxicity due to its oxidative bioactivation by CYP2E1 and FAD-containing monooxygenases, leading to S-oxide (TAASO), and then to its chemically reactive S, S-dioxide (TAASO_2_), generating ROS, depletion of antioxidant enzymes, and injury to hepatocytes and cholangiocytes (Hajovsky et al. 2012; Wallace et al. 2015). These reactive metabolites promote lipid/protein damage and hepatocyte death, which may trigger an inflammatory response after multiple administrations. Oxidative stress, cell death, and inflammatory mediators are the main stimuli for HSC activation and further collagen deposition, which may culminate in liver fibrosis and cirrhosis processes when chronically administered (intraperitoneal administrations or in drinking water) (Wallace et al. 2015). Although this chemical is rarely used in isolation as a model for HCC induction due to a long latency for HCC burden, a recent study showed that 34-week chronic TAA administration can be used to induce frank HCC development together with well-defined cholangiocarcinoma in male Lewis rats (Hu et al. 2024).

In general, medium-term exposure (8–30 weeks) to CCl_4_ or TAA did not result in the establishment of a fibrosis-associated hepatocarcinogenesis model (Weber et al. 2003; Wallace et al. 2015). For a frank development of preneoplastic and neoplastic lesions into a fibrotic scenario, two-stage models using DEN-initiated animals and chronic CCl_4_ or TAA administrations are more suitable.

## Comparison of previous two-stage rat models using DEN/CCl_4_ or DEN/TAA regimens

The use of two-stage rodent models in which the initiation of hepatocarcinogenesis is induced by DEN (infant/neonate and adult models), followed by a promoting phase with repeated TAA or CCl_4_ administrations, is often used to cause a high HCC burden (tumor incidence and number) into a fibrosis/cirrhosis microenvironment in a reduced time compared to conventional single agent models (Table 1) (Cho and Jang 1993; Park et al. 2001; Lim 2003; Uehara et al. 2013; 2014; Kimura et al. 2013; Luo et al. 2013; Furtado et al. 2014; Marrone et al. 2016; Romualdo et al. 2017, 2018; Memon et al. 2020; Hora et al. 2021). The adherence to chemical-promoting cycles increases the incidence of preneoplastic FAH, HCA, and HCC in rats and mice treated with CCl_4_/TAA_,_ while increasing the multiplicity and size of glutathione transferase placental form (GST-P)-positive preneoplastic lesions (FAH and nodules) and tumors in rats treated with TAA or CCl_4_ (Cho and Jang 1993; Uehara et al. 2013, 2014; Furtado et al. 2014; Romualdo et al. 2017, 2018; Memon et al., 2020). Thus, we revisited ourprevious findings from two 25-week fibrosis/cirrhosis-associated rat hepatocarcinogenesis models by administering successive CCl_4_ or TAA injections in DEN-initiated adult male Wistar rats, mimicking the chronic liver injury as seen in human liver disease (Furtado et al. 2014; Romualdo et al. 2017). In this comparison, we used the immunoreactivity for GST-P as a preneoplastic and neoplastic biomarker associated with rat hepatocarcinogenesis (Tsuda et al. 2003; Sakai and Muramatsu, 2007; Kimura et al. 2013).

**Table 1:** Rodent models of fibrosis/cirrhosis-associated with hepatocarcinogenesis induced by carbon tetrachloride (CCl4) or thioacetamide (TAA) regimen in animals initiated with diethylnitrosamine (DEN)

References	Animal/Age	Model	Treatment/Euthanasia	Outcomes
Hora et al. (2021)	Male Wistar rats (320–340 g)	Adult	DEN (200 mg/kg b.w., i.p., single dose) and cycles of TAA (200 mg/kg b.wt., i.p., with 1 or 3 days of interval doses for two months) and euthanized at 1 or 3–6 months after TAA regimen	↑ Liver cirrhosis and HCC (100%) (6 months) ↑ Serum ALT, AST and ALP levels ↑ MDA levels and ↓ GSH level and catalase activity
Memon et al. (2020)	Male and female C57BL/6 mice (14.5-day-old)	Infant	DEN [20 mg/kg (first dose), 30 mg/kg (second dose) and 50 mg/kg (six doses) b.w., i.p.] and after 1 week received TAA (300 mg/kg b.w. i.p. twice a week for 4 or 8 weeks) and euthanized at week 24	↓ Body weight gain and ↑ Liver weight (TAA 4 and 8 weeks) ↑ Development of liver tumors (100%,) and ↑ Serum AST and ALT levels (TAA 4 and 8 weeks) ↑ Hepatocyte proliferation (PCNA) and apoptosis (TUNEL) (TAA 4 and 8 weeks) ↑ Collagen deposition, Cox-2 protein levels and α-SMA immunoreactivity (TAA 4 and 8 weeks) ↓ Survival ratio (85%, TAA 8 weeks) l
Romulado et al. (2018)	Male and female C3H/HeJ mice (2 weeks old)	Infant	DEN (10 or 50 mg/Kg. b.w., i.p., single dose) and six weeks later CCl4 (0.25 to 1.50 μL/g b.wt., i.p. 3 weekly doses) and euthanized at week 17	↓ Body weight gain and ↑ liver weight (males and females, 10 and 50 mg) ↑ Serum ALT and AST levels (males, 10 and 50 mg, and females, 50 mg) ↑ Development of preneoplastic AHF and HCA (males and females, mainly in 10 mg) and a few HCC (males, 10 mg) ↑ Hepatocyte proliferation (Ki-67) and apoptosis (caspase 3) ↑ Collagen deposition, α-SMA, and CD68 (mainly in males, 10 and 50) ↑ Hepatic IL-6 and COX-2 levels (males in 10 and 50 mg) Collagen deposition, α-SMA and CD68 (mainly in male, 10 and 50)
Romualdo et al. (2017)	Male Wistar rats (6 weeks old)	Adult	DEN (200 mg/kg b.w., i.p., single dose) and two weeks late cycles of TAA (200 mg/kg b.wt., i.p., twice a week during three weeks, and each cycle was followed by one week without treatment) and euthanized at week 25	↓ Body weight gain and ↑ Liver weight ↑ Serum ALT and AST levels, Collagen deposition, and Hepatocyte cell proliferation (Ki-67) ↑ Development of preneoplastic GST-P positive lesions, HCA, HCC, and CCA ↑ Oxidative stress (LOOH levels) and ↓ Antioxidant defense (Catalase, GST, and GSH-Px activities) ↑ MMP-2 and 9 activities ↑ Hepatic mRNA levels of pro-inflammatory genes (Lsp1, Ctse, Ccl21b, C1qa, C1qb, and Ccl21b) ↑ Hepatic mRNA levels of extracellular matrix genes (Col1α1, Col1α2, Timp1, Timp2) and regulators of collagen synthesis (Anxa 2, Dcn and Lgals3 bp) ↓ Hepatic mRNA levels of antioxidant enzymes genes (Gpx1, Gstm3, Cat)
Marrone et al. (2016)	Male B6C3F1 mice (2 weeks old)	Infant	DEN (1 mg/kg b.w., i.p., single dose) and six weeks later CCl4 (0.2 ml/kg,b.w., i.p., twice a week for 14 weeks) and euthanized at week 22	↑ miRNA levels associated with cell proliferation and cell cycle deregulation, apoptosis, inflammation, oxidative stress, and fibrosis and cirrhosis ↑ mRNA levels of Il10, Osm, F4/80, and S100a4 (Fps1) (inflammation-related genes) and Vim, Mmp9, and Tgfb1 (fibrosis-related genes)
Furtado et al. (2014)	Male Wistar rats (6 weeks old)	Adult	DEN [200 mg/kg b.w., i.p., single dose) and and two weeks later CCl4 (0.5 ml/kg b.w. i.g., once a week from week 2–10 and 1.0 ml/kg b.w. from week 11–24) and euthanized at week 25	↓ Body weight gain and ↑ liver weight ↑ Serum ALT levels, collagen deposition, and hepatocyte cell proliferation (PCNA) ↑ Development of preneoplastic GST-P positive lesions, HCA and HCC ↑ Hepatic mRNA collagen 1 and 3
Luo et al., (2013)	Male BalbC mice (6 weeks old)	Adult	DEN (1 mg/kg b.w., i.p., single dose) and six weeks later CCl4 (20%, 0.1 ml/10 g b.w., i.g., twice a week from week 2–18) and euthanized at week 20	↑ Liver cirrhosis (100%) ↑ Development of preneoplastic lesions and HCC (40%) ↑ Development of preneoplastic GGT-positive foci ↑mRNA levels of c-myc, N-ras and p53
Kimura et al. (2013)	Male F344/NSlc rats (6 weeks old)	Adult	DEN (200 mg/kg b.w., i.p., single dose) and two weeks late drinking water containing 0.02% TAA or 6 weeks, 70% partial hepatectomy at week 3 after DEN treatment, and euthanized at week 8	↓ Body weight gain and ↑ liver weight ↑Number and area of preneoplastic lesions GST-P-positive foci ↑ Hepatocyte proliferation (Ki-67) and apoptosis (caspase 3) ↑ p16Ink4a expression in GST-P-positive foci
Uehara et al. (2013, 2014)	Male B6C3F1 mice (2 weeks old)	Infant	DEN (1 mg/kg b.w., i.p., single dose) and six weeks later CCl4 (0.2 ml/kg,b.w., i.p., twice a week for 9 or 14 weeks) trom week 2–18) and euthanized at week 22	↑ Liver weight and serum ALT levels ↑ Development of preneoplastic lesions, HCD (100%) and HCC (50%) Development of preneoplastic lesions and HCC (40%) ↑ 4-HNE-adducted proteins and 8-oxo-dG adducts ↑ Immunostaining of F4/80, CD68, CD11b, MHC2, and αSMA ↓ mRNA levels of Ogg1, Apex, and Polβ (DNA repair genes) ↑ mRNA levels of Ccr1, Ccr5, Ccl3, and Cx3cr1 (chemokines genes) and Timp-1 (matrix remodeling gene) ↑ mRNA levels of Afp, H19, and Bex1 (fetal genes), Prom1 and Epcam (stem cell markers genes) and p16 (cell-cycle inhibitor gene)
Park et al. 2001), Lim (2003)	Male Fischer 344 (6 weeks old)	Adult	DEN (200 mg/kg b.w., i.p., single dose) followed by PB (0.05%) for 1 week and TAA (0.03% in drinking water for 39 weeks).and euthanized 9, 20, 30 and 40 weeks after DEN application	↑ Liver cirrhosis (20,30 and 40 weeks) ↑ Development of HCA (30 weeks) and HCC (40 weeks) Gankyrin expression (liver fibrosis stage), degradation of pRB (cirrhosis stage) and inactivation of p16INK4A and p15INK4B (hypermethylation) (tumor stage)
Cho and Jang (1993)	Male Sprague–Dawley rats (1-day-old)	Infant	DEN (15 mg/kg b.w., i.p., single dose) and three weeks later CCl4 (33% in 0.2 ml mineral oil., i.p. twice a week from week 3–12) and euthanized at week 12	↑ Liver cirrhosis (35%) and incomplete septation (75%) ↑ Development of preneoplastic lesions and nodules GST-P positive ↑ Hepatocyte proliferation (BrdU) and apoptosis (TUNEL)

**Fig. 1:**
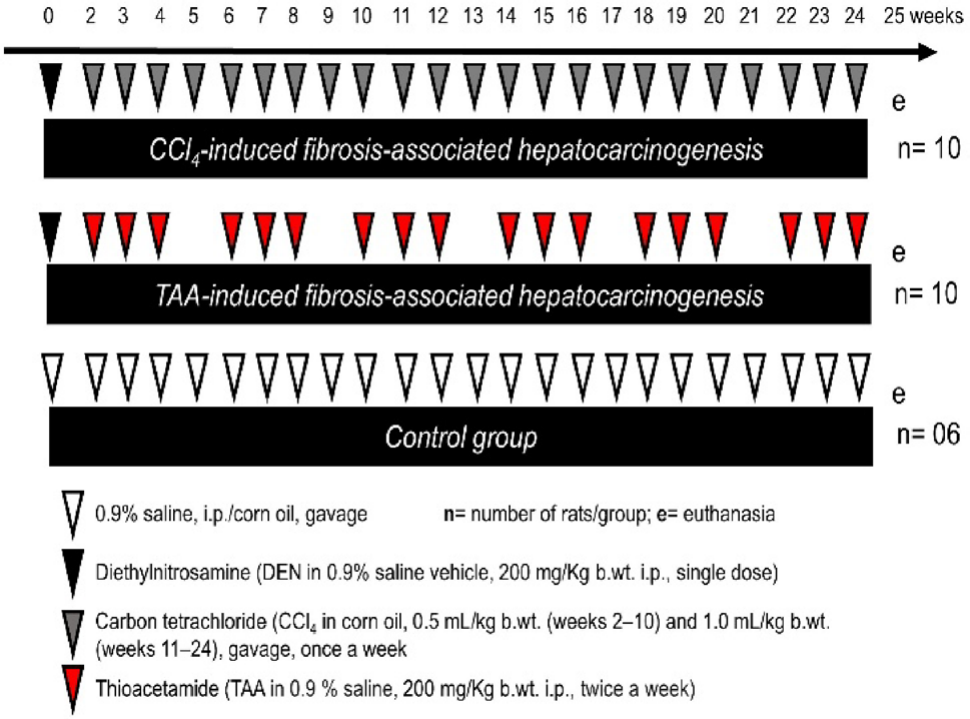
Experimental design. Adult male Wistar rats were submitted to two different regimens of cirrhosis-induced hepatocarcinogenesis. Animals received a single intraperitoneal (i.p.) injection of diethylnitrosamine (DEN, 200 mg/Kg b.wt.) or 0.9% saline vehicle. After 2 weeks, rats were kept on two different fibrogenic regimens with intraperitoneal injections of carbon tetrachloride (CCl_4_, 0.5–1.0 mL/Kg b.wt.), thioacetamide (TAA, 200 mg/Kg b.wt.), or 0.9% saline vehicle. All animals were euthanized after 25 weeks of a single DEN administration. Created in Microsoft PowerPoint

Afp, Alpha fetoprotein, ALT, alanine aminotransferase, Apex, Apurinic/apyrimidinic endonuclease, AST, aspartate aminotransferase, ALP, Alkaline phosphatase, Bex1, Brain expressed X-linked, protein 1, BrDU, Bromodeoxyuridine, b.w., Body weight, CCA, Cholangiocarcinoma, Ccl3, Chemokine (C–C motif) ligand 3, Ccr1 and 5, Chemokine (C–C motif) receptor 1 and 5, Cx3cr1, Chemokine (C-X3-C) receptor 1, Epcam, Epithelial cell adhesion molecule, GGT, y-glutamyltransferase, GSH-Px, Glutathione peroxidase, GST, glutathione-S-transferase, GST-P, Glutathione S-transferase placental form, 4-HNE, 4-hydroxynonenal, H19, H19 fetal liver mRNA, HCA, Hepatocellular adenoma, HCC, Hepatocellular carcinoma, i.p., intraperitoneal injection, i.g., intragastrical administration, LOOH, lipid hydroperoxide, MMP-2 and 9, Matrix metalloproteinases 2 and 9, Ogg1, 8-oxoguanine DNA-glycosylase pRB, nuclear phosphoprotein of retinoblastoma tumor suppressor gene. p16INK4, INK4 family of cyclin dependent kinase inhibitors, PCNA, Proliferating cell nuclear antigen, levels, Polβ, Polymerase (DNA directed), beta1 Prom1, Prominin 1Timp-1, 2, tissue inhibitor of metalloproteinases-1 and 2, TUNEL, TdT-mediated dUTP nick-end labeling, α-SMA, Alpha-smooth muscle actin. ↓, decrease ↑, increase;

To DEN/TAA and DEN/CCl_4_ regimen comparison (Fig. 1), six-week-old male Wistar rats were randomly allocated into three groups: Groups 1 and 2 (n = 10 each/group) were given a single intraperitoneal (i.p.) injection of DEN (200 mg/kg b.w., diluted in 0.9% NaCl). Two weeks later, group 1 received CCl_4_ 10% solution in corn oil by gavage once a week (0.5 ml/kg b.w. from weeks 2–10 and then at 1.0 ml/kg b.w. from weeks 11–25) (Furtado et al. 2014). Group 2 received TAA (200 mg/kg b.w., diluted in 0.9% NaCl) by i.p. injections twice a week for three weeks, and each cycle was followed by one week without treatment (Romualdo et al. 2017). Group 3 (n = 06) received only vehicle administration. These groups were reanalyzed from previous animal studies approved by the Local Ethics Committee for Animal Use (CEUA) from Institute of Biosciences of Botucatu/UNESP/Brazil (protocol number 588 and 1073).

At week 25, the animals were euthanized, and blood samples were collected for serum alanine aminotransferase (ALT) level analysis (Ozer et al. 2008), and the liver was excised, macroscopically analyzed, and weighed. Liver sections were used for histopathological analysis after hematoxylin–eosin (HE) staining, collagen analysis after Picrosirius red staining, and GST-P or proliferating cell nuclear antigen (PCNA) using immunohistochemical reactions. Liver sections stained for Sirius red were also evaluated for fibrosis/cirrhosis, classified on a score of 0–4 (Goodman 2007). Preneoplastic AFH, hyperplastic nodules, and neoplastic lesions were identified and classified in HE-stained liver sections according to criteria previously published (Thoolen et al. 2012).

**Fig. 2:**
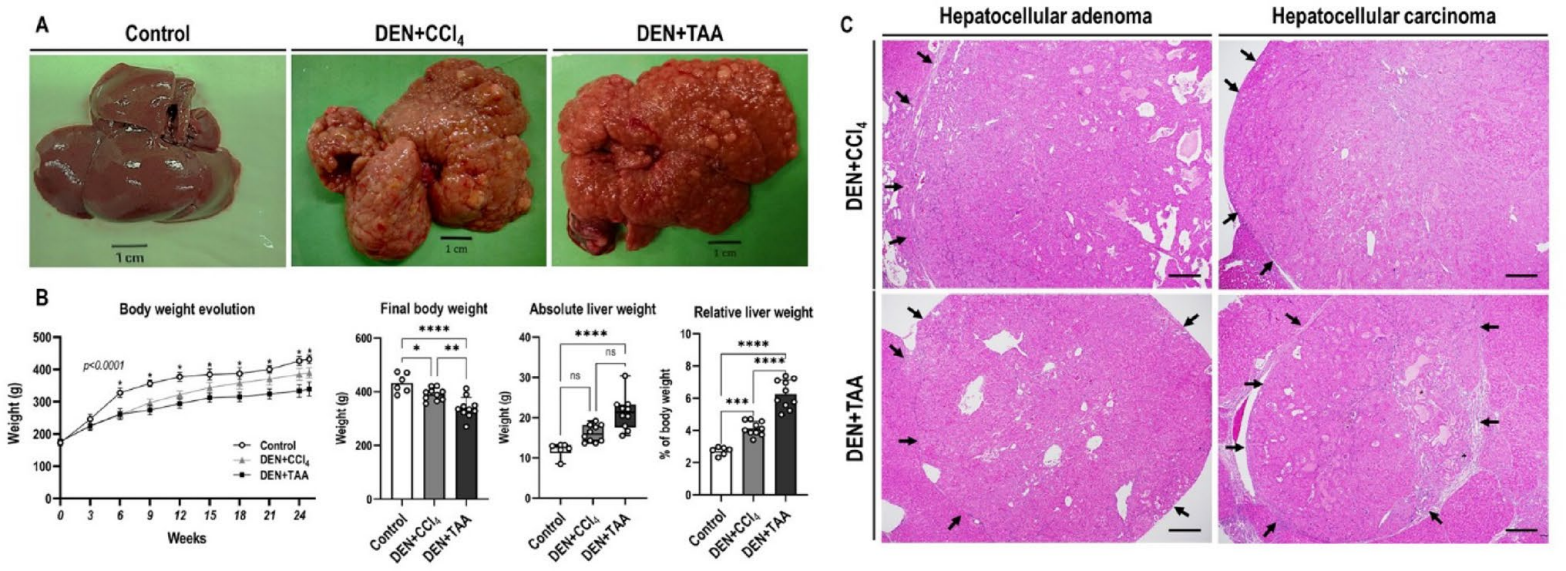
General findings. **A** Macroscopic view of the liver from male rats submitted to the two-stage cirrhosis-associated hepatocarcinogenesis models. **B** Chronic effects of carbon tetrachloride (CCl_4_) or thioacetamide (TAA) regimens in diethylnitrosamine (DEN)-initiated rats on the body weight evolution, final body weight, and absolute and relative liver weights. **C** Representative photomicrographs of hematoxylin–eosin (HE)-stained sections of hepatic lesions (hepatocellular adenoma or carcinoma, arrows) (10 × objective). Data are presented as mean ± standard deviation or median and max. and min., and considered statistically significant when *p* < 0.05. One-way ANOVA or Kruskal Wallis test followed by Tukey test. Statistical analyses and graphics were performed using GraphPad Prism software 6.0 (GraphPad, San Diego, CA, USA). * *p* < 0.05, ** *p* < 0.01, *** *p* < 0.001, **** *p* < 0.0001. Bar = 1 cm (A) or 100 µm (C)

**Fig. 3:**
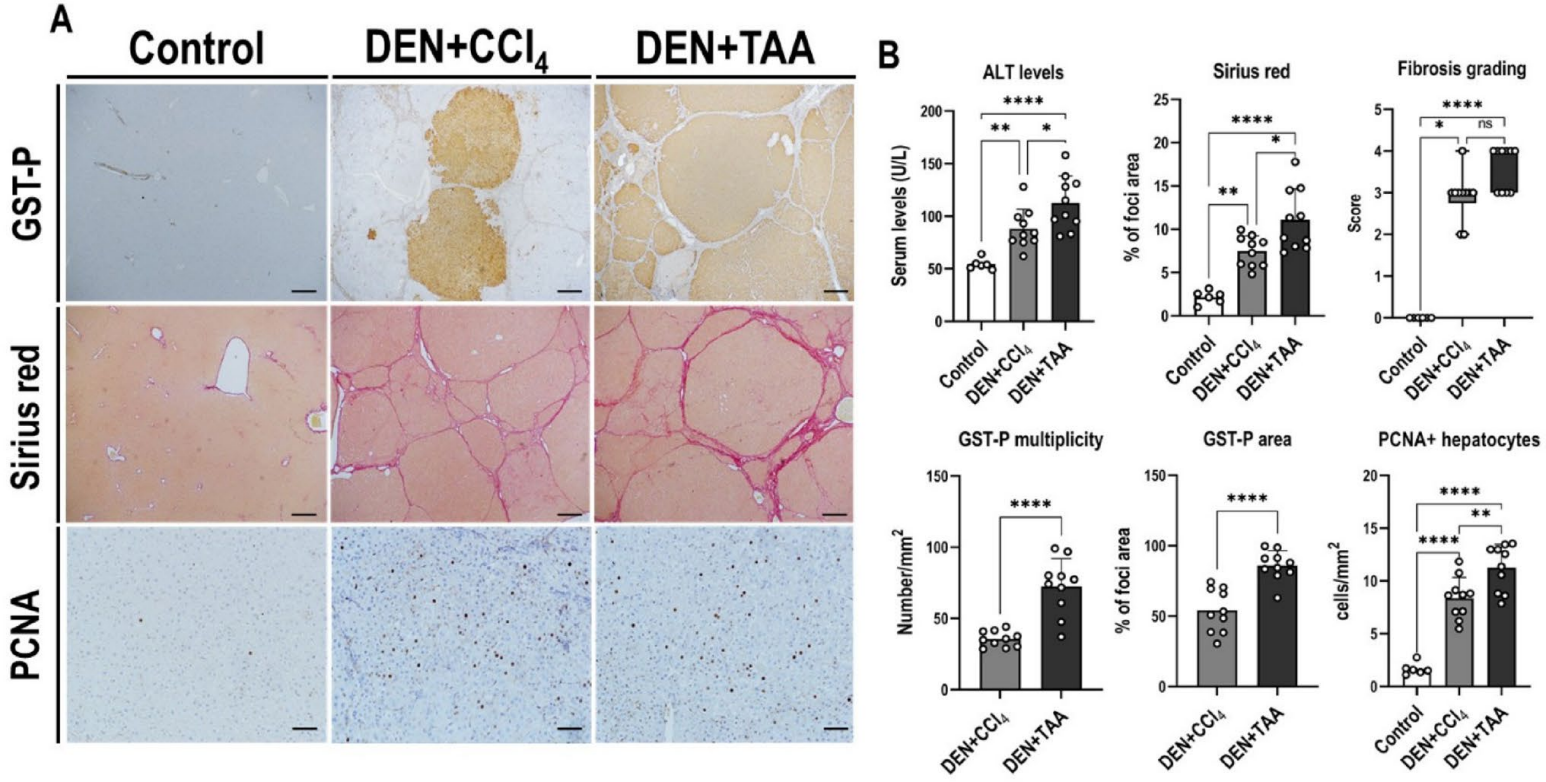
General findings. **A** Representative photomicrographs of Sirius red-stained and glutathione S-transferase placental form (GST-P) and proliferating cell nuclear antigen (PCNA)-immunoreacted hepatic sections. Bar = 100 µm (GST-P and Sirius red) or 50 µm (PCNA). **B** Determination of serum ALT levels and morphometric assessment of fibrosis-related parameters (hepatic collagen content and fibrosis severity score), hepatic preneoplastic GTS-P foci and nodules occurrence and relative liver area (%), and number of PCNA-positive hepatocyte/liver area. Data are presented as mean ± standard deviation, or median and max. and min., and considered statistically significant when *p* < 0.05. One-way ANOVA or Kruskal Wallis test followed by Tukey test. Statistical analyses and graphics were performed using GraphPad Prism software 6.0 (GraphPad, San Diego, CA, USA). * *p* < 0.05, ** *p* < 0.01, **** *p* < 0.0001). Photomicrographs were obtained in a microscopy Olympus BX65 accoupled in an Olympus cellSens software (Olympus Corporation, Shinjuku, Japan)

As expected, macroscopic findings of liver damage were observed in rats exposed to both TAA or CCl_4_ regimens, such as rough surface aspect (Fig. 2A), while both protocols reduced body weight gain and final body weight (*p* < 0.0001) (Fig. 2B). Representative photomicrographs of HCA and HCC are depicted in Fig. 2C. Both protocols led to a significant increase in absolute and relative liver weight (*p* < 0.0001) (Fig. 2B). Representative photomicrographs of liver sections stained for picrosirius, GST-P and PCNA immunoreactivities are depicted in Fig. 3A. In DEN/CCl_4_ or TAA, collagen deposition (*p* < 0.0001), and fibrosis grading (*p* < 0.0001) (Fig. 3B), compared to the control group, which is consistent with cirrhotic scarring onset. It is noteworthy that the DEN/TAA regimen enhanced the serum ALT levels (*p* = 0.0278), the hepatic collagen content (*p* = 0.0101), both number (*p* < 0.0001) and relative area (*p* < 0.0001) of GST-P-positive FAH and hyperplastic nodules, and the number of PCNA-positive hepatocytes (*p* = 0.0054), in comparison to DEN/CCl_4_ group (Fig. 3B), suggesting that the TAA protocol might accelerate liver disease progression and carcinogenesis.

The comparative morphological and biochemical findings indicate that DEN-initiated and chronically injured with TAA or CCl_4_ rats developed liver injury and fibrosis/cirrhosis with high serum ALT levels, hyperplastic nodules, and collagen deposition. In addition, both models caused a pronounced development of preneoplastic lesions (FAH and hyperplastic nodules) positive for GST-P enzyme and considered to be suitable precursors of HCC (Tsuda et al. 2003; Sakai and Muramatsu 2007). Importantly, there are no studies in parallel comparing two different medium-term (8–30 weeks) protocols of chemically induced fibrosis/cirrhosis in DEN-initiated rats. Although these two models yielded liver cirrhosis (80–100%, scores 3 and 4) with no mortality during CCl_4_ /TAA administrations, overt ascites were not observed until 25 weeks. Therefore, both protocols showed very similar variability for most parameters evaluated. However, the DEN/TAA regimen was clearly superior for inducing hepatocellular lesions positive for GST-P expression and mainly tumors/per rat (6.80 ± 3.65 vs. 3.80 ± 2.49, p = 0.045). This difference between groups could potentially be resolved by modifying the CCl_4_ protocol. In terms of experimental design and considering that 70–90% of human HCC cases emerge in a cirrhotic background, the repeated administration of these fibrogenic agents is recommended to induce chronic liver damage that resembles human disease and pathology (Yanguas et al. 2016; Faccioli et al. 2022; Romualdo et al. 2021, 2023). Besides, a few studies have evaluated gene expression and protein profiles during progression from liver fibrosis/cirrhosis and hepatocarcinogenesis; the two-stage rodent models have shown some similarities with human liver disease (Luo et al. 2013; Marrone et al. 2016; Romualdo et al. 2017; Sun et al. 2019; Hora et al. 2021; Petrenko et al. 2024).

As expected, both two-stage 25-week protocols led to the frequent occurrence of hepatocellular preneoplastic and neoplastic lesions in the extensive fibrosis background, as depicted by the GST-P immunostaining and histopathological analysis, and assessment of the hepatic collagen content and fibrosis score, respectively. However, it is noteworthy that the TAA regimen enhanced the emergence and growth of hepatic neoplasms and the number and area of preneoplastic AHF and nodules positive for GST-P (Fig. 3B), as well as hepatic collagen deposition, compared to the CCl_4_ regimen. Indeed, it has been previously reported that TAA might induce more aggressive damage to the hepatic architecture and fibrosis when compared to CCl_4_ and acetaminophen regimens (Wallace et al. 2015). As exploring the hepatic fibrotic/cirrhotic milieu is essential for a clearer understanding of liver cancer occurrence, since up to 80–90% of HCC cases emerge in such conditions (Sung et al. 2021; Ginès et al. 2021; Rumgay et al. 2022; Huang et al. 2023; Zamani et al. 2025). Our findings on these two classical rat models highlight major differences at morphological level that should be considered in future studies, as previously reported by Singh et al. (2023) In general, the DEN/TAA regimen was able to resemble late-stage hepatocarcinogenesis, displaying a prominent fibrotic background and prominent emergence of hepatic tumor, which lead us to a further molecular strategy for unveil potential drivers of HCC with “omics” strategies and then, establish a parallel with molecular findings of the corresponding disease in patients.

TAA is a highly toxic chemical compound that is mostly metabolized in the liver by hepatocytes, being defined as an effective drug for hepatotoxicity studies by the 50 s’, as reviewed by Hajovsky et al. (2012). Usually, the toxicological effects of TAA are mostly driven by the oxidative bioactivation process of the main enzyme CYP2E1 in hepatocytes, leading to the synthesis of sulphur-based metabolites (TASO and TASO_2_) that might induce protein carbonylation and DNA damage, and initiate hepatocytes to liver carcinogenesis in an oxidative stress-related mechanism (Hajosky et al., 2012)_._ The chronic exposure to TAA leads to the synthesis of ROS and mitochondrial impairment, leading to the activation of HSC, regarded as quiescent hepatic parenchymal cells that, under specific inflammatory stimuli, change their phenotype into myofibroblast-like cells that are responsible for the synthesis of the largest collagen pool during liver fibrosis/cirrhosis progression (Lee et al. 2015; Yanguas et al. 2016; Hammerich and Tacke 2023). In addition, the continuous exposure to TAA promotes the activation of central transcription factors of inflammatory-oxidative pathways (*e.g.,* NF-κB and NRF2) that might induce the recruitment of immune cells and induce the exhaustion of antioxidant mechanisms—mostly related to glutathione-related enzyme mechanisms—of hepatocytes, triggering the hepatic inflammatory microenvironment that enables liver cancer progression (Dwivedi and Jena 2020; Teck et al. 2004; Petrenko et al. 2024). Although the CCl_4_ is widely considered a hepatocarcinogen, this fibrotic agent generally does not act as an initiator (which causes direct DNA mutations) but it acts as a powerful tumor promoter for liver carcinogenesis in an oxidative stress-related mechanism (Romualdo et al. 2018; Cohen et al. 2023).

## Conclusions and future perspectives

In this narrative review, we highlight the translational value of classic chemically-induced cirrhosis-associated hepatocarcinogenesis models in rats and mice, as a useful tool for recapitulating trending features of human liver disease. HCC is the most common primary liver cancer and the third leading cause of disease-related death, presenting a poor prognosis (Huang et al. 2023; Zamani et al. 2025). In addition, the most traditional therapies available for HCC, such as liver resection or transplantation, percutaneous ethanol injection, and radiofrequency ablation, lose their effectiveness with disease progression (Huang et al. 2023; Zamani et al. 2025). Thus, preclinical studies evaluating preventive strategies given during the early and late stages of fibrosis/cirrhosis-associated hepatocarcinogenesis are important for HCC control, including dietary and natural antioxidant and anti-inflammatory agents, vitamins, and trace elements. Thus, HCC can be prevented by early detection and public health measures that reduce exposure to known risk factors for this malignancy. Several natural bioactive compounds (BCs) and non-conventional drugs have been tested as prophylactic or therapeutic strategies to prevent hepatocarcinogenesis in different DEN + CCl_4_ and DEN + TAA regimens (Table 2), including α-lipoic acid (Fuji et al., 2013), vitamin D3 (Goto et al. 2022), simvastatin (Elleithi et al. 2023), carbenoxolone (El-Zehery et al. 2024); allyl isothiocyanate (Bronzato et al. 2025), hesperidin (Mahmoud et al. 2017), coffee bioactive coumpounds (Furtado et al. 2014, Romualdo et al., 2022), ginsenoside Rk1 (Wu et al. 2024), pantoprazole (Jin et al., 2024).

**Table 2:** Prophylactic (Pro) or therapeutic (Ther) actions of drug or purified bioactive compounds against liver fibrosis/cirrhosisassociated with hepatocarcinogenesis in rodent studies

Study	Animal/Age	Protocol	Treatment (dose/period)	Outcomes (liver/serum)
*DEN + TAA models*				
Fujii et al. (2013)	Male F344/NSlc rats (5-week-old)	DEN (200 mg/kg b.w., single i.p. injection) plus TAA (0.02% in drinking water for 6 weeks) plus 70% partial hepatectomy 3 weeks after DEN injection	α-LA (0.2% in drinking water for 6 weeks- Pro model)	↓ Number and size of preneoplastic GST-P-positive lesions ↓ Number of hepatocytes in proliferation (PCNA-positive cells) and apoptosis (TUNEL-positive cells) ↓ Macrophages (positive or CD133, Cox 2 and HO-1) and lymphocytes T (CD3-positive) ↑TUNEL and active Casp 8 and 9-positive cells inside of GST-P lesions ↓ mRNA Col1a1, Mmp2, Ptgs2, Tgfb2, Cx3cl1, Cxcl10 and Serpine1 levels
Goto et al. (2022)	Male Wistar rats (6-week old)	DEN (200 mg/kg b.w., single i.p. injection) plus TAA (200 mg/kg b.w., i.p., injection 3 times a week/ five cycles for 24 weeks)	VD3 (5000 and 10,000 IU/kg diet) for week 2–25 weeks during TAA regimen – Pro model)	↓ Collagen deposition and p65 (Nk-kβ) protein levels ↑ CAT and GSH-Px activities ↑ VD receptor protein levels, ↓ Number of preneoplastic GST-P and CK 8/18-positive lesions and HCA
Elleithi et al. (2023)	Male Sprague Dawley (8–9-week old)	DEN (200 mg/kg b.w., single i.p. injection) plus TAA (200 mg/kg b.w., i.p. injection, 2 times a week for 16 weeks)	SIM (10 mg/kg, i.g. administrations for 3 weeks after TAA regimen- Ther model)	↓ Relative liver weight, liver injury and fibrosis and TGF-β1 ↓ Serum AFP and total bilirubin levels and ↑ albumin levels ↓ Fibrosis and necroinflammation ↑ Apoptosis and mRNA of Casp8 and ↓ mRNA Bcl2 levels
El-Zehery et al. (2024)	Male Sprague Dawley (8–9-week old)	DEN (200 mg/kg b.w., single i.p. injection) plus TAA (200 mg/kg b.w., i.p. injection, 2 times a week for 16 weeks)	CBX (20 mg/kg, i.p., the last 4 weeks during TAA regimen -Pro model- or 7 weeks after TAA regimen – Ther model)	↓ Relative liver weight, liver injury and fibrosis and TGF-β1 (immunoreactivity) ↓ Serum AFP, ALT, AST and total bilirubin levels ↑ mRNA of TRAIL, TRAILR2, casp 3, 8, and 9 levels ↑ Apoptosis and ↑ Bcl-2 (mRNA and immunoreactivity) levels
Bronzato et al. (2025)	Male C57Bl6/j mice (2-week old)	DEN (25 mg/kg b.w., two i.p. doses) plus TAA (200 mg/kg b.w., i.p., 3 times a week/five cycles for 24 weeks)	AITC (2 mg/kg bw., i.g., 5 days/week for weeks 8–13 during TAA regimen – Pro model)	↓ Number of macrophages (CD68-positive) ↑ Antioxidant activity
*DEN + CCL*_4_ *models*				
Furtado et al. (2014)	Male Wistar rats (6-week old)	DEN (200 mg/kg b.w., single i.p. injection) plus CCL4 (0.5 ml/kg b.w. from weeks 2–10 and 1.0 ml/kg b.w. from weeks 11–24, once a week for 24 weeks)	0.1% CAF in drinking water for 5 days/week from week 2–25—Pro model)	↓ Collagen deposition and mRNA expression of collagen I ↓ Size and % area of GST-P- positive preneoplastic lesions ↑ bax protein levels
Mahmoud et al. (2017)	Male Wistar rats (6–7 week old)	DEN (200 mg/kg b.w., single i.p. injection) plus CCL_4_ (3 ml/kg b.w., s.c. injection, once∖week for 16 weeks)	Hesperidin (50 or 100 mg/kg b.w., i.g. administration, daily for 18 weeks- Pro model)	↓ Serum ALT, AST, ALP, LDH and bilirubin levels ↓ Serum APF and CEA levels ↓ Liver injury, collagen deposition and proliferation (PCNA) ↓ MDA levels and ↑ SOD, GPx, GSH and GST ↓TGF-β1 protein and Smad3 phosphorylation levels ↓ Serum TNF-α and NF-κB levels ↑ Nrf2 (gene and protein expression) and HO-1 and PPARγ (protein expression)
Romualdo et al. (2021)	Male C3H/HeJ mice (2-week old)	DEN (25 mg/kg b.w., single i.p. injection) plus CCL4 (0.25–1.50 μl/g b.wt., 5 days/week for weeks 8–18)	CAF (50 mg/kg b.wt.), CAF + TRI (50 and 25 mg/kg b.wt./day); CAF and CGA (50 and 25 mg/kg b.wt./day); CAF,TRI and CGA treatments (50, 25 and 25 mg/kg b.wt./day. All treatments 5 days/week for weeks 7–17- (Pro model)	↓ Incidence and number of preneoplastic lesions (CAF + TRI + CGA) ↓ Proliferation (Ki-67) in preneoplastic lesions (CAF + TRI + CGA) ↓ Collagen deposition, CD68 (macrophages) and p65 (Nk-kβ), α-SMA (stellate cell) and IL-17 protein levels ↓ TBARS levels and ↑ SOD and GSH-Px activities ↑ Tumor suppressors miR-144-3p, miR-376a-3p and antifibrotic miR-15b-5p
Wu et al. (2024)	Male C57BL/6 mice (-week old)	DEN (10 mg/kg b.w., single i.p. injection) plus CCL_4_ (0.20 mlg/kg b.wt., twice a week for 17 weeks)	L-Rk1 (30 or 60 mg/kg b.w., i.g. administration, daily for 8 weeks- Ther model)	↓ Relative liver weight, liver injury and fibrosis/cirrhosis ↓ Serum and liver tissue of ALT and AST levels and AFP levels (gene and protein) ↑ AMPK/mTOR protein activation and autophagy (LC3-II protein) and ↓ Bcl2 protein
Jin et al. (2024)	Male C57BL/6 mice (2-week old)	DEN (10 mg/kg b.w., single i.p. injection) plus CCL_4_ (0.50 mlg/kg b.wt., twice a week for 14 weeks)	PPZ (1, 2, 5 or 10 mg/kg b.w., I.p. injections, twice daily or 12 weeks during CCL_4_ regimen- Prop model-, or for 6 weeks after CCL_4_ regimen- Ther model)	↓ Number and size of HCC ↓ mRNA of IL1, CXCL1, CXCL5, CXCL10, CCL2, CCL6, CCL7, and CCL20 (inflammatory genes)

AIT, allyl isothiocyanate; AFP, alpha fetoprotein; ALP, alkaline phosphatase; ALT, alanine aminotransferase; AMPK, AMP-activated protein kinase; AST, aspartate aminotransferase; Bcl-2, B-cell lymphoma 2; b.w, body weight; CAF, caffeine, CCL/CXCL, chemokines; CAT, catalase; CBX, carbenoxolone; CCL4, carbon tetrachloride; CEA, carcinoembryonic antigen; CK, cytokeratin; Cox2, cyclooxygenase 2; DEN, diethylnitrosamine; GCA, chlorogenic acid; GPx, glutathione peroxidase; GSH, reduced glutathione; GST, glutathione-S-transferase; GST-P, glutathione S-transferase, placental form; HCA, hepatocellular adenoma; HCC, Hepatocellular carcinoma; HO-1, hemeoxygenase-1; IL, interleukin; i.p, Intraperitoneal injection; α-LA, α-lipoic acid; LDH, lactate dehydrogenase; L-Rk1, Ginsenoside Rk1; MDA, malondialdehyde; mTOR, mammalian target of rapamycin; miR, miRNA; Nrf2, nuclear factor (erythroid-derived 2)-like 2; PCNA, proliferating cell nuclear antigen; PPARγ, peroxisome proliferator activated receptor gamma; PPZ, pantoprazole; s.c., subcutaneous; SOD, superoxide dismutase; TAA, thioacetamide; TBARS, Thiobarbituric acid reactive substances; TGF-β1, transforming growth factor-β1; TNF-α, tumor necrosis factor alpha; TRAIL, tumor necrosis factor-related apoptosis-inducing ligand; TRAILR, TRAIL receptor; TRI, trigonelline; SIM, simvastatin; VD3, vitamin D3. ↑, increase; ↓ reduce

Considering the new cutting-edge high-resolution methods availability (omics), a wider and integrative investigation into these models are necessary, since only a minority of the reports on two-stage chemical-induced models explore such parameters. Thus, the use of multi-omics integration analysis (single cell and spatial transcriptomics, proteomics, metabolomics) in essential for elucidating the mechanisms of these models. For example, the use of single-cell transcriptomics (scRNA-seq) technology in liver fibrosis-associated HCC studies are essential to reveals cellular heterogeneity, identifying specific HSC and immune cells subtypes, mapping intercellular signaling pathways driving progression from fibrosis to HCC, understanding of therapy resistance, and using in personalized medicine and targeted therapy for HCC (Chung et al. 2022; Zang et al., 2022). In rodent in liver fibrosis models, scRNA-seq analysis has been recently introduced. Deng et al. (2025), using scRNA-seq analysis, have shown that in TAA or CCl_4_ -induced fibrosis models, HSCs, Kupffer cells, and T-cell subsets exhibit distinct regulatory patterns and dynamic remodeling processes in these models. These findings highlight the heterogeneity of immune responses and extracellular matrix (ECM) remodeling in different fibrosis models such as TAA or CCl_4_-induced, BDL, and NASH, providing important insights into the complex mechanisms underlying liver fibrosis. Liu et al. (2025), using assay for transposase-accessible chromatin sequencing (ATAC-seq), RNA-seq, and scRNA-seq, showed the CCl_4_-liver fibrosis process may be associated with the acute accumulation of lipids during liver damage repair, accompanied by increased histone acetylation throughout the entire period and inhibition of mitochondrial electron transport chain (ETC) activity in the early stage of CCl_4_-induced liver injury. Although these two-stage models resemble the corresponding human disease with various molecular and morphological outcomes (Graphical Abstract, created in BioRender—https://www.figurelabs.ai/.), some limitations are clear in our revisited findings: (1) we used only one protocol of CCl_4_ and TAA application in DEN-initiated rats; (2) we did not include additional control groups (treated with DEN only, CCl_4_ only and TAA only) to distinguish contribution of each of the chemicals; (3) their high systemic toxicity and animal mortality can limit their use in long-term interventions and they do not recapitulate MASH, obesity, or insulin resistance as seen in human MASLD/MASH-driven HCC (Romualdo et al. 2023; Wu et al. 2023).

## Data Availability

The datasets used in the review study are available from the corresponding author on reasonable request.
